# Dietary restrictions in healing among speakers of Iquito, an endangered language of the Peruvian Amazon

**DOI:** 10.1186/1746-4269-7-20

**Published:** 2011-07-11

**Authors:** Kevin A Jernigan

**Affiliations:** 1 Department of Ethnobotany, University of Alaska, Fairbanks, Kuskokwim Campus, 201 Akiak Dr., Bethel, AK, USA

**Keywords:** Iquito, Ethnomedicine, Dietary Taboos, Peru, Endangered Languages

## Abstract

**Background:**

Ethnobotanical research was carried out with speakers of Iquito, a critically endangered Amazonian language of the Zaparoan family. The study focused on the concept of "dieting" (***siyan++ni ***in Iquito), a practice involving prohibitions considered necessary to the healing process. These restrictions include: 1) foods and activities that can exacerbate illness, 2) environmental influences that conflict with some methods of healing (e.g. steam baths or enemas) and 3) foods and activities forbidden by the spirits of certain powerful medicinal plants. The study tested the following hypotheses: H1 - Each restriction will correlate with specific elements in illness explanatory models and H2 - Illnesses whose explanatory models have personalistic elements will show a greater number and variety of restrictions than those based on naturalistic reasoning.

**Methods:**

The work was carried out in 2009 and 2010 in the Alto Nanay region of Peru. In structured interviews, informants gave explanatory models for illness categories, including etiologies, pathophysiologies, treatments and dietary restrictions necessary for 49 illnesses. Seventeen botanical vouchers for species said to have powerful spirits that require diets were also collected.

**Results:**

All restrictions found correspond to some aspect of illness explanatory models. Thirty-five percent match up with specific illness etiologies, 53% correspond to particular pathophysiologies, 18% correspond with overall seriousness of the illness and 18% are only found with particular forms of treatment. Diets based on personalistic reasoning have a significantly higher average number of restrictions than those based on naturalistic reasoning.

**Conclusions:**

Dieting plays a central role in healing among Iquito speakers. Specific prohibitions can be explained in terms of specific aspects of illness etiologies, pathophysiologies and treatments. Although the Amazonian literature contains few studies focusing on dietary proscriptions over a wide range of illnesses, some specific restrictions reported here do correspond with trends seen in other Amazonian societies, particularly those related to sympathetic reasoning and for magical and spiritual uses of plants.

## Background

### Dietary Proscriptions in Healing

Dietary restrictions accompanying the healing process have been reported from geographically widespread locations, including Africa [1,2], Europe [3], North America [1], Southeast Asia [4,5], and South America [6-8]. The connection between diet and medicine forms an important part of the classical and modern humoral traditions of India, China, ancient Greece and medieval Europe [9-12]. Some authors [13,14] have also pointed out the importance of dietary context for understanding the physiological effects of medicinal plants from a biomedical perspective. A number of recent review articles [15-17] have also treated dietary taboos in a broader social context.

In the Amazonian case, the ethnomedical literature [18-23] has tended to discuss dietary restrictions in the context of spiritual traditions related to ayahuasca and other psychoactive plants, as well as their role in learning how to heal in that tradition [19,21]. However, the healing systems of many Amazonian societies [24-27] rely on cures of both a physical and spiritual nature. Attitudes toward medicinal plants also reflect this duality. On one hand, plants are thought to have physical effects on the body. On the other, they are believed to possess spirits that can actively participate in the healing process. Physical cures are often common knowledge that any adult possesses, while spiritual cures tend to be the province of specialists. Lenaerts argues, based on his work with the Ashéninka and other Amazonian societies [25,28], that these two forms of healing are not simply parallel systems coexisting in the same place. Rather, the spiritual level is the one of real power and efficacy, while the physical is merely useful for relieving symptoms. Other authors [8,29,30] have made similar observations in other Amazonian societies.

This paper presents the results of research on the relationship between illness explanatory models (EMs) [31] and corresponding restrictions on diet and activities for speakers of Iquito, a critically endangered [32] language of the Peruvian Amazon. The explanatory model is a framework for describing a cognitive model of how illness works in terms of five components: 1) etiology, 2) onset of symptoms, 3) pathophysiology, 4) prognosis and 5) treatment [31]. This framework allows for a detailed examination of the rationales for dietary restrictions within the logic of a given ethnomedical system. The first hypothesis of this research is: **H1 - Each restriction will correlate in a systematic way with one or more elements of the illness explanatory models**. In other words, one might find that fish with sharp teeth are never eaten for illnesses involving internal bleeding, or that patients given enemas must always avoid rain and cold.

The Iquito are no exception to the general Amazonian trend of plurality of medical reasoning, making reference to spiritual, emotional, humoral and contamination-related illness etiologies, among others. However, some of these etiologies appear to have been adopted fairly recently. Study participants said that their ancestors attributed spiritual causes to any illness that did not have an obvious physical cause. Taking that claim as a cue, and following the observation of various authors [25,28-30] in favor of the primacy of spiritual over naturalistic healing modalities in other Amazonian societies, the second research hypothesis is: **H2 - Illnesses whose explanatory models have personalistic elements will show a greater number and variety of restrictions than those based on naturalistic reasoning**. For example, one would expect to find greater restrictions when a plant spirit or witchcraft is believed to be involved, than for cures based on humoral reasoning.

### History of the Iquito Language

**Figure 1:**
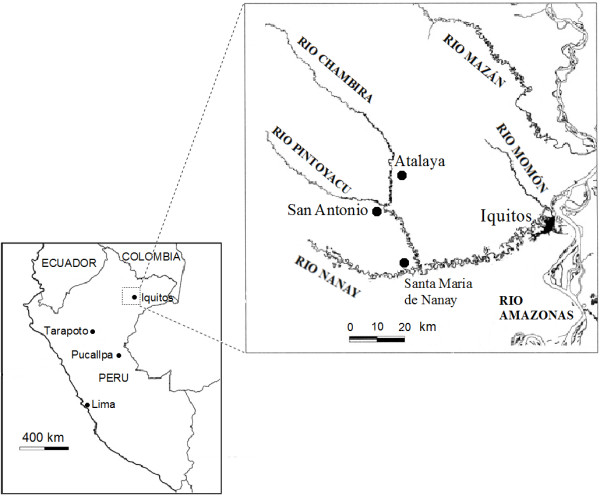
**Map of the study area**.

Iquito is a language of the Zaparoan family, spoken by roughly twenty people, living in two villages in the Alto Nanay region of the north-eastern Amazon (Figure 1). According to early Spanish colonial reports, the language once extended over the area between the Tigre, Mazán and Amazonas rivers. The Iquito moved away from the major rivers from the 17^th ^to the 19^th ^centuries to avoid mission settlements and slave raids. By the early 20^th ^century, they started to form larger settlements downriver, including the village of San Antonio, on the Pintoyacu river, where this study took place [33,34].

Although *mestizo *(mixed native and European) settlers arrived with the rubber boom in the 1920s [34], the Iquito were essentially monolingual through the 1930s. With the death of the Iquito *curaca *(traditional leader) in 1944, the *patrones *(rubber bosses) consolidated their power, controlling the native population with a system of debt peonage. The newcomers brought a very negative attitude toward the Iquito language and discouraged its use, sometimes with violent means. By the end of the 1950s, most adults were already bilingual in Spanish and many children did not learn Iquito at all [33].

The 1950s and 60s saw another influx of *mestizo *settlers. Mixed marriages were common, and children in those families mostly did not learn Iquito. At the same time, new epidemics of flu, malaria and other infectious diseases killed many older monolingual speakers. By the 1990s, Spanish had largely replaced Iquito in everyday interactions.

Currently, none of the 20 or so speakers remaining is less than 50 years old. Negative feeling towards the language still exists, although it has diminished. In the last decade, linguists have been working on an Iquito-Spanish dictionary [35] and on bilingual educational materials. The research described in this paper is part of a larger project representing the first study of Iquito ethnobotany and will hopefully contribute in some modest way to recent efforts [33,35-37] to document the language.

## Methods

The study took place from 2009 to 2010, principally in the village of San Antonio Pintoyacu, in the Alto Nanay region of Loreto, Peru. Authorization for conducting research was obtained from the Peruvian Ministry of Agriculture (no. 324-2009-INENA-DGFFS-DGEFFS). Upon arriving in San Antonio, a meeting took place to discuss the study goals and the research plan, and to give community members a chance to ask questions and voice any concerns they might have. All participants gave verbal prior informed consent (PIC), and the research followed ethical guidelines adopted by the American Anthropological Association [38].

**Figure 2:**
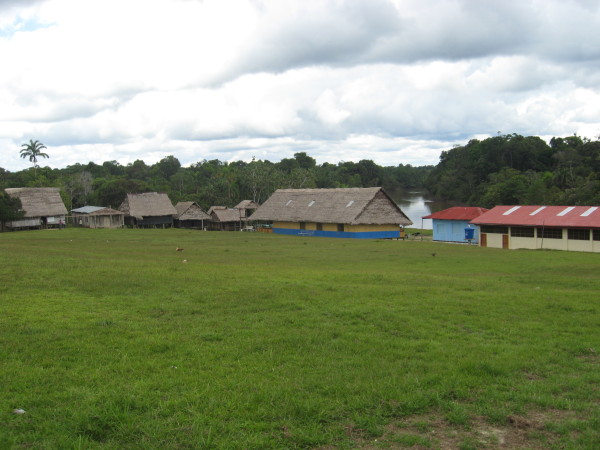
**The community of Atalaya, Alto Nanay region, Loreto, Peru**.

Of approximately twenty remaining Iquito speakers, six were recruited in San Antonio to participate in the study. Four of those provided the bulk of the data described in this article, while two others helped to further clarify and expand upon the data. Two non-speakers provided additional assistance with collection of botanical voucher specimens in the community of Atalaya (Figure 2), on the Chambira river. Five participants are bilingual in Spanish, while one is the last semi-monolingual speaker of Iquito. Although a larger sample would be ideal in a study of this kind, the very small number of fluent speakers who are also experts in medicinal plants greatly limited the participant pool. Some speakers did not value ethnobotanical knowledge due to years of repression of their indigenous identity [34]. Interviews were carried out primarily in Spanish, although much Iquito vocabulary relevant to illnesses and medicinal plants was collected as well. Note that Iquito plant and illness names provided in the text are shown in bold and italics, while Spanish names are given in italics. The Iquito character "+" represents a close central unrounded vowel.

In structured interviews, participants named 49 illness categories. Explanatory models were collected for each category, including etiologies, pathophysiologies and treatments. Informants described the types of dietary restrictions required for each treatment, including the foods and activities avoided and the length of time. They also explained the reason for the diet, whether due to a plant spirit, the method of ingestion, or to avoid making the illness worse.

Seventeen voucher specimens were collected for plant species requiring special diets. These correspond to 14 botanical families. All vouchers were deposited in the herbarium of the Universidad Nacional de la Amazonía UNAP, in Iquitos, Peru.

## Results and Discussion

### An Overview of Illness Categories and Explanatory Models

Table 1 shows the 49 illness categories informants mentioned in the interviews. They cover a range of internal and external complaints as well as some magical conditions. A few local illness terms and culture-bound syndromes require explanation. ***Aquíraja ***(*choque de aire *in Spanish) involves diarrhea and vomiting and is thought to come from being struck by a strong wind carrying bad spirits or harmful environmental influences. *Corazonada *is a dangerously strong heartbeat that comes from arguing with one's spouse. *Empacho *is a digestive illness said to be caused by bad diet, involving intestinal swelling, headache, vomiting and diarrhea. ***Isíicu ***(*sarna *in Spanish) refers to a variety of skin infections. Informants recognized several types, including ***ácusana ***('red') ***isíicu***, characterized by red, itchy spots, ***musutina ***('white') ***isíicu***, involving white spots that are not painful, and ***s+ríca***, which causes white, itchy patches [35]. ***Múcuaay+ itúuja ***- literally "rainbow burn" is a rash caused by exposure to a rainbow, the sun or mist. *Pulsario *is a worm that lives in the lower abdomen and grows bigger with emotional stress. *Saladera *involves persistent bad luck, especially in hunting. *Sobreparto *is an illness women may suffer after giving birth that involves diarrhea, body pain and fever. *Tabardillo *is a high fever caused by bathing too quickly after working in the sun.

**Table 1:** Illnesses and Corresponding Diets

ILLNESS	EXPLANATORY MODEL	DIET
DIGESTIVE ILLNESSES		

***aquíraja ***(see text)	bad air, spirits	wind

colic	temperature	spicy, cold

constipation	bad food (too dry, certain fruits)	none

diarrhea (simple)	temperature, dirty water, bad food	spicy, cold, heat, soup

dysentery	temperature, dirty water	spicy, pork, cold, heat, soup, oil

*empacho *(see text)	bad food, temperature	pork, cold, soup

parasites	bad food, walking barefoot	sweet

stomach ache	temperature	spicy, cold

BITES AND STINGS		

bullet ant bite	poisoning, mechanical	spicy

scorpion sting	poisoning, mechanical	spicy, toothed animals, sex, cold, heat

snake bite	poisoning, mechanical	spicy, sour, pork, toothed fish and animals, sex, cold, heat

stingray sting	poisoning, mechanical	sex, cold

EXTERNAL PROBLEMS		

acariasis	parasites, bad hygiene	bad blood

burns	physical	spicy, sex, heat

***isíicu ***(see text)	bad blood, sympathetic magic, bad hygiene	bad blood

leishmaniasis	*microbios *(see text), spirits	spicy, pork, toothed fish and animals, bad blood, sex, cold, heat

mouth sores	bad blood, sympathetic magic	none

***múcuaay+ itúuja ***(see text)	rainbow, sun, fog, bad air	spicy, heat

pus-filled boils	bad blood, physical	spicy, bad blood, cold

wounds	mechanical	spicy, cold, heat

FEVER RELATED		

bronchitis	bad air, temperature	spicy, pork, sex, cold

cholera	bad air, spirits, bad water	sweet, spicy, pork, soup, sex, cold, heat

fever	temperature	cold, heat

flu	bad air, spirits	spicy, sex, cold, heat

malaria	bad water, bad food, bad blood, mosquitoes, sexually transmitted, *microbios *(see text)	spicy, pork, sex, cold, heat

measles	bad air, spirits	spicy, sour, toothed fish and animals, soup, sex, cold, heat, oil

*tabardillo *(see text)	temperature	cold, heat

whooping cough	bad air, spirits	sweet, spicy, sour, pork, toothed fish and animals, soup, sex, cold, heat

OTHER ILLNESSES		

anemia	bad blood, lack of blood, *microbios *(see text), bad hygiene	sweet

*corazonada *(see text)	emotional	none

ear infections	temperature	cold, wind

eye problems	mechanical, temperature, bad air	spicy, heat, spider webs in the eyes

dislocations	mechanical	spicy, exertion

fractures	mechanical	spicy, exertion

general weakness	lack of blood, malnutrition	none

head ache	temperature, bad blood	none

haemorrhages	temperature	sweet, spicy, sour, toothed fish and animals, soup, sex, heat, exertion

hernia	physical	spicy, sex, cold, exertion

insomnia (in children)	temperature, lack of blood	none

kidney problems	temperature, bad water	spicy, pork, toothed fish and animals, alcohol

liver problems	bad or strong food and drink, temperature	spicy, pork, toothed fish and animals alcohol

love problems	emotional	none

*pulsario *(see text)	emotional	spicy, sour, toothed fish and animals, sex

rheumatism	temperature	spicy, sex, cold, heat

*saladera *(see text)	personalistic	hunting animals in forest, being seen by people

*sobreparto *(see text)	mechanical	cold

temper tantrum (in children)	emotional	none

uterine problems	temperature, mechanical, *microbios *(see text)	spicy, sour, toothed fish and animals, soup, sex, cold, heat, alcohol

witchcraft	personalistic	spicy, sour, pork, toothed fish and animals, bad blood, sex, cold

As already noted, informants said the old Iquito attributed spiritual causes to any illness without an obvious physical origin. Infectious diseases in particular, such as measles and whooping cough were said to have a spirit like a person (***niatíja ***- literally "mother" in Iquito). When a wave of epidemics came with the first *mestizo *settlers of the 1920s, people said the spirits flew through the air like angels searching for people to infect. The Iquito were not unique in this regard, Chaumeil mentions a similar belief among the neighboring Yagua [29], as does Bellier [39] for the Maihuna. Another type of personalistic etiology involves witchcraft. Witches are said to attack by a variety of means, including causing an invisible phlegm-like substance to lodge in the victim's body, producing severe pain.

Currently, witchcraft is still taken seriously in San Antonio, but belief in illness spirits has fallen out of favor. Study participants were more inclined to invoke humoral explanations, including excessive temperature and ***aquíraja ***- "bad air or wind." These EMs are broadly similar to humoral theories of health found in many parts of Central and South America [11,13]. Scholars have debated whether these ideas originated independently in the New World [40,41], or were simply borrowed from the Spanish [11]. The illness category ***s'+m+siini ***- "rheumatism" is a good example of humoral reasoning. When a person is exposed to night air, rain, wind or frigid bath water, the cold can stay in the bones and joints, causing pain and discomfort. Heat from the sun or from cooked foods and beverages can cause ***ípanaca ***- 'fever,' and enter bodily organs, potentially causing them damage. Moving too rapidly from one temperature extreme to another can also be harmful. Some informants say kidney problems can arise from taking a cold bath too soon after working in the hot sun. The rapid change causes the blood to become viscous and not circulate properly, which also harms internal organs.

Study collaborators attributed some illnesses to bad water or food. Standing water is commonly associated with ***tan+'+ca ***- "malaria." It is considered harmful because animals defecate in it, or because it contains "*microbios*." The later term is ambiguous, as it is used for small visible organisms such as insect larvae as well as for microbes in the biomedical sense. Simple diarrhea (without blood or mucous) is blamed on eating greasy foods on an empty stomach, while intestinal parasites are said to come from consuming sweet fruits like ripe plantains and *caimito *(*Pouteria caimito*). The old Iquito took purges such as ***ácuta ***(*Banisteriopsis caapi*) and *curarina *(*Potalia amara*) often to cleanse impurities from the body, but such treatments have fallen out of use in recent times.

Some illnesses result from contamination of the blood. Healthy blood is considered key to maintaining overall good health. Lenaerts [25] has noted similar beliefs among the Ashéninka. One of the most common illnesses attributed to bad blood is ***pisáqui ***- "pus-filled boils." Contamination can come from eating the meat of certain animals like tapirs, whose blood is dark and considered to be dangerously strong. Blood-related illnesses and skin problems in general are also said to come from eating fish with spotted patterns. This is an example of sympathetic reasoning, whereby foods are thought to transfer particular qualities to people who eat them. Similarly, babies are susceptible to ***m+'+riaaca ***- "mouth sores," when a breastfeeding mother consumes spotted fish. Some people said biting flies and mosquitoes can contaminate the blood because they also bite dogs or wild animals such as snakes and transfer that foreign blood a person's body.

A few illness etiologies do not fit easily into any of the above categories. Getting dirt in an eye from running into spider webs in the forest causes **carijáquica - "**eye pain." Intestinal parasites are thought to infect people who walk barefoot, through the soles of the feet. Many informants noted that doctors told them malaria comes from mosquito bites, although some were skeptical about that explanation. Some illnesses have emotional causes, including the culturally bound syndromes *corazonada *and *pulsario *mentioned above.

### The Role of Dieting in Healing

Avoiding certain foods and activities is often a necessary part of the healing process for Iquito speakers of San Antonio. Informants used the Spanish term *dietar *or the Iquito ***siyan++ni ***- "to diet" to describe this process. All collaborators related examples of people who became more ill or died from failing to follow expected restrictions.

Three main reasons for restrictions were given: 1) some foods and activities can exacerbate an illness, 2) some methods of administration (i.e. steam baths or enemas) leave a patient susceptible to harm from hot or cold environmental influences, 3) some healing plants possess spirits that require the patient to avoid certain foods or activities. In the first category, the diet is explained in terms of the etiology and pathophysiology of the illness. In the second and third, the restrictions are required to avoid conflicting with the treatment itself.

The following section describes common classes of dietary restrictions mentioned for two or more illnesses. These are grouped into categories and presented in terms of illness explanatory models.

#### Spicy Foods

Avoidance of spicy foods, in particular, ***nap'+qui ***- "hot pepper" (*Capsicum frutescens*), is the most common dietary proscription, mentioned for 63% of illnesses (Table 1). This restriction applies especially for bites, stings, infectious diseases and illnesses involving internal organs. Pepper forms an important part of the traditional Iquito diet in such dishes as ***jicuriáaca***, a soup made with manioc beer and meat or fish. However, it is also considered to be potentially quite harmful for people in a weakened state due to illness. One informant noted that pepper warms the blood and can burn internal organs, including the stomach, liver and kidneys.

#### Environmental Influences

Restrictions on environmental influences are found for 61% of total illnesses, making this the second most common category. Rain, night air and cold foods are said to exacerbate illnesses involving fevers, gas, diarrhea and serious bites or sores. Hot influences, including strong sun, cooked foods and being too near a cooking fire interfere with recovering from fevers, burns and wounds. Wind is dangerous for patients suffering from the culture-bound syndrome ***aquíraja ***- "bad air," or from ear infections.

Sometimes temperature extremes must be avoided even when they were not the original cause of the illness. For example, cold can enter a sore while a person is bathing and make it worse. Some methods of curing also leave a person vulnerable to harm from excessive temperatures. Exposure to cold foods, beverages or environmental influences after taking an enema or steam bath can cause intestinal swelling or death. It is interesting that some very similar humoral reasoning can be found in other parts of the world. For example, among Malays, both hot and cold influences are dangerous for fevers while cold influences are bad for digestive illness [42].

#### Meat and Fish

Restrictions on meat and fish are also quite common, found for 45% of total illnesses. Pork is considered one of the most potentially harmful because of its high fat content and because pigs eat garbage. This restriction does not seem to correspond with any particular category of illness. However, it does correspond to total number of diet items for a given illness. In other words, the more restrictions a given illness has, the more likely pork will be one of them. The average number of restrictions when pork is included is 5.3. The average for illnesses not including pork as a restriction is 2.4. Results of a two-tailed Mann Whitney U test [43] give a P value of < 0.001, which is considered a highly statistically significant difference, by standard criteria.

Through sympathetic reasoning, the meat of fish and mammals with sharp teeth is said to be harmful for those recovering from problems with internal organs or serious bites or stings. Those foods are also forbidden to anyone suffering from witchcraft, since sharp teeth invoke the magical darts witches are thought to use to harm their victims. In his study of dietary restrictions in Búzios island, São Paolo State, Brazil, Begossi [44]has also noted that carnivorous fish are avoided by people recovering from illness.

As previously mentioned, the meat of tapirs, peccaries and fish with spots is associated with bad blood. Those foods are harmful to anyone suffering from a skin infection.

#### Sex

Sexual abstinence is considered important for recovering from 37% of total illnesses, particularly for bites, stings, and some illnesses involving fevers or problems with internal organs. One informant maintained that sex causes the blood to circulate excessively and to penetrate the intestines. He also noted that it is dangerous because the soul momentarily escapes from the body. Another noted that women and men have differing humors, and also likened the prohibition to similar ones pertaining to meat.

#### Sour

Sour foods, including some fruits and ***itíniija ***- "manioc beer" are forbidden while recuperating from 14% of total illnesses. This prohibition does not appear to correspond to any particular kind of illness, but instead correlates with the total number of dietary restrictions. Results of a two-tailed Mann Whitney U test show diets including sour foods have a significantly higher average number of restrictions than those that do not.

#### Soup

Meat and fish prepared in a soup must be avoided for a number of illnesses involving bodily secretions such as blood, diarrhea and phlegm (14% of total illnesses). The logic behind this prohibition appears to be sympathetic.

#### Sweet

People with parasites or diarrhea (10% of total illnesses) must not consume sweet foods, including many fruits and manioc beer that has not fully fermented. Such foods are said to feed parasites and cause them to increase in number.

#### Alcohol

Distilled spirits are incompatible with 8% of total illnesses, particularly those affecting internal organs, especially the kidney, liver and uterus. One informant said that alcohol can burn these organs.

#### Oil

Oily foods are contraindicated for 8% of illnesses. This prohibition does not correspond to any particular illness category, but instead appears to correlate with the total number of dietary restrictions. Results of a two-tailed Mann Whitney U test show diets including oily foods have a significantly higher average number of restrictions than those that do not.

#### Exertion

Physical activity interferes with recovery from a few illnesses (8% of the total) with obvious mechanical etiologies, such as hernia and fractures.

#### Diets Required By Plant Spirits

In traditional Iquito belief, all plants have spirits, but some particularly powerful medicinal species are said to be *celoso *- "jealous," because they require anyone who ingests them to follow rigorous diets. Seventeen vouchers were collected for species in this category (Table 2).

**Table 2:** Plants with spirits that require diets

SPECIES	VCH.	COMMON NAME	USE	DIET
ANACARDIACEAE				

*Spondias mombin *L.	416	***m+tiíja nap+níja***	uterine problems	spicy, sour, pork, toothed fish and animals, bad blood, sex, cold, heat

APOCYNACEAE				

*Tabernaemontana macrocalyx *Müll. Arg.	403	*uchu sanango*	rheumatism, body pain, chronic sores	salt, sweet, sour, pork, toothed fish and animals, bad blood, soup, sex, heat, wind, oil, alcohol, manioc, exertion

ARACEAE				

*Dieffenbachia *sp.	402	***sapatíqui***	altars the effects of ayahuasca	salt, sweet, spicy, sour, pork, toothed fish and animals, bad blood, soup, sex, heat, wind, alcohol, manioc, exertion

*Dieffenbachia smithii *Croat	401	***áqu+siiti***	altars the effects of ayahuasca	salt, sweet, spicy, sour, pork, toothed fish and animals, bad blood, soup, sex, heat, wind, alcohol, manioc, exertion

BIGNONIACEAE				

*Mansoa alliacea *A.H. Gentry	335	***m+'+s+y+***	fever, flu, *saladera *(see text)	spicy, sour, pork, toothed fish and animals, bad blood, sex, cold, heat

CELASTRACEAE				

*Maytenus macrocarpa *(Ruiz & Pav.) Briq.	391	*chuchuhuasi*	rheumatism	salt, sweet, spicy, sour, pork, toothed fish and animals, bad blood, soup, sex, cold, heat, wind, oil, alcohol, manioc, exertion

CLUSIACEAE				

*Tovomita cephalostigma *Vesque	385	***suníina***	learning medicine	salt, sweet, spicy, sour, pork, toothed fish and animals, bad blood, soup, sex, cold, heat, wind, oil, alcohol, manioc, exertion, isolation

EUPHORBIACEAE				

*Hura crepitans *L.	415	*catahua*	treating witchcraft	salt, sweet, spicy, sour, pork, toothed fish and animals, bad blood, soup, sex, cold, heat, wind, oil, alcohol, manioc, exertion

MALPIGHIACEAE				

*Banisteriopsis caapi *(Spruce ex Griseb.) C.V. Morton	426	***ácuta***	cleans the stomach, visionary, learning medicine	salt, sweet, spicy, sour, pork, toothed fish and animals, bad blood, soup, sex, heat, wind, alcohol, manioc, exertion

MENISPERMACEAE				

*Abuta grandifolia *Aubl.	382	*motelo sanango*	rheumatism, chills	salt, sweet, spicy, sour, pork, toothed fish and animals, bad blood, soup, sex, cold, heat, wind, oil, alcohol, manioc, exertion

MORACEAE				

*Ficus insipida *Willd.	328	*ojé*	parasites	salt, sweet, spicy, sour, pork, toothed fish and animals, bad blood, sex, cold, heat, oil

OLACACEAE				

*Minquartia *sp.	374	*huacapú*	anemia	salt, sweet, spicy, sour, pork, toothed fish and animals, bad blood, soup, sex, cold, heat, wind, oil, alcohol, manioc, exertion

POACEAE				

*Paspalum *sp.	363	*gramalote*	ear ache	other natural or pharmaceutical remedies

PHYTOLACCACEAE				

*Petiveria alliacea *L.	393	*mucura*	*saladera*	being seen by other people, hunting animals in forest

SOLANACEAE				

*Brunfelsia grandiflora *D. Don	413	*sanango*	rheumatism, body pain, *saladera*	salt, sweet, spicy, sour, pork, toothed fish and animals, bad blood, soup, sex, cold, heat, wind, oil, alcohol, manioc, exertion, isolation

*Brugmansia flaveolens *(Humb. & Bonpl. ex Willd.) Bercht. & C. Presl	358	***isúuna***	learning medicine	salt, sweet, spicy, sour, pork, toothed fish and animals, bad blood, soup, sex, cold, heat, wind, oil, alcohol, manioc, exertion, isolation

ZINGIBERACEAE				

*Renealmia alpinia *(Rottb.) Maas	428	***naquijina mirija***	*saladera*	being seen by other people, hunting animals in the forest

When plants with "jealous" spirits are given to treat illness, diets tend to be more extensive than what is required simply to avoid exacerbating illnesses. In fact, two restrictions, manioc and salt, are only found for plants in this group. Oral ingestion generally carries the most restrictions, particularly when it is an infusion or decoction in water. When plants are prepared in alcohol, diets are often much looser, but potency is also weakened.

Other plants in this group were used to learn how to be a ***paanáana ***- a specialist in spiritual healing. This process required months of isolation, strict sexual abstinence and avoidance of any strong foods, so that the spirit of the plant would appear and teach the dieter how to heal. In the most extreme cases, dieters were said to eat only the leaves of **asúraaja **(*Manihot esculenta*) and ***ám++ca ***(*Phytolacca rivinoides*). Informants maintained that their ancestors learned medicine mainly from ***isúuna ***(*Brugmansia suaveolens*) and ***ácuta ***(*Banisteriopsis caapi*). Interestingly, the primary admixture plants for *Banisteriopsis caapi *in San Antonio are *Dieffenbachia *(called ***m++m'++ti ***in Iquito or '*chacruna*' in Spanish). Only one other literature reference (López Vinatea 2000) could be found mentioning this genus as an ayahuasca admixture. Study participants said other magical plants were learned after contact with mestizos and the arrival of colonists from other places. One informant, for example, said she learned from Lamas Quechua settlers that *quión *(*Hedychium *sp.) is capable of showing a person in dreams what kind of healer he can become.

The results of breaking diets with "jealous" plants are often more serious than simply making the illness worse. They are said to punish or bewitch people who break the diet. Some consequences are relatively minor. For example, if a person consumes sugar too soon after taking *sanango *(*Brunfelsia grandiflora*), he will get white spots on the head. Similarly, one informant said the old Iquito did not used to diet properly when taking ***ácuta ***(*Banisteriopsis caapi*), and thus, often suffered skin rashes. Later, healers from outside taught people to avoid sex and eat only very bland foods for a period of time after taking ayahuasca. Other punishments are much more serious. Breaking a diet with *ojé *(*Ficus insipida*) will punish a diet breaker by making him go crazy as the tree's white resinous sap comes out of all bodily orifices.

#### Other

A few restrictions apply only to a single illness. A person with an eye infection must avoid running into spider webs in the forest. Dirt in webs is considered harmful to the eye and is regarded as a possible cause of infections. To cure the culture bound syndrome *saladera*, the victim must go far into the forest and bathe with a mixture of urine and strong smelling plants such as *mucura *(*Petiveria alliacea*) and ***m+'+s+y+ ***(*Mansoa alliacea*). He must avoid being seen by anyone else during this process and refrain from hunting any animal he encounters there.

### Addressing the Hypotheses

The first hypothesis, that each restriction will correlate in a systematic way with one or more elements of the illness explanatory models, is supported by the data. Seventeen distinct dietary restrictions were mentioned two or more times in the interviews. All correlate to some aspect of the illness EMs. Six (35%), (wind, heat, cold, bad blood, sweet foods and exertion) correspond to particular illness etiologies. Nine (53%), (spicy foods, heat, cold, toothed animals and fish, sex, soup, sweet foods, alcohol and exertion) correspond to specific illness pathophysiologies. Two (12%), (heat and cold), correspond to treatments administered as enemas or steam baths. Three prohibitions (18%), (oily foods, sour foods and pork) correspond only to the overall seriousness of an illness. Three restrictions (18%), (salt, manioc and isolation) are only found in cases of treatments involving powerful plant spirits.

The data also clearly support the second hypothesis that diets based on explanatory models with personalistic reasoning will show a differing number of and kinds of restrictions than those based on naturalistic reasoning. The former category includes all diets required by plant spirits and those for magical illnesses such as witchcraft and *saladera*. The average number of prohibitions for personalistic EMs is 9.4, while the average for naturalistic EMs is 3.1. Results of a two-tailed Mann Whitney U test show a highly statistically significant difference.

## Conclusions

Restrictions of diet and activities play a central role in healing among Iquito speakers, and their ethnomedical system cannot be properly understood without taking them into account. Such prohibitions can be explained in terms of specific aspects of illnesses and their treatments. In general, restrictions will be more extensive for illnesses whose cause or treatment has a strong spiritual dimension.

Some prohibitions found among the Iquito correspond with trends seen in other Amazonian societies. Some authors have noted the importance of restrictions for magical and spiritual uses of plants, among *mestizos *[19,23] and other indigenous societies [8]. Valadeau *et al*. [8] report that the Yanesha employ similar sympathetic reasoning, for example, in prohibiting meat from animals with sharp teeth to anyone suffering snake bite. The Ashéninka [25] share analogous ideas about contamination from eating certain species of animals.

Commonalities with other Amazonian societies raise the question of which ideas found in San Antonio have been borrowed. Some of the most salient medicinal plants participants mentioned, such as *sanango *(*Brunfelsia grandiflora*) and *ojé *(*Ficus insipida*) are only known by names of obvious Spanish or Quechua origin. Also, many important illness categories, including culture-bound syndromes such as *saladera *have no Iquito name. Future research could involve comparative studies in neighboring indigenous or *mestizo *communities to examine what aspects of Iquito diet beliefs appear to be unique to them and which appear to be pan-Amazonian.

## Competing interests

The author declares that they have no competing interests.
